# 2019 JAPAN Critical Limb Ischemia Database (JCLIMB) Annual Report

**DOI:** 10.3400/avd.ar.22-00072

**Published:** 2022-09-25

**Authors:** The Japanese Society for Vascular Surgery JCLIMB Committee, NCD JCLIMB Analytical Team

**Keywords:** arterial occlusive disease, leg ischemia, peripheral arterial disease (PAD), CLI, annual report

## Abstract

Since 2013, the Japanese Society for Vascular Surgery has started the project of nationwide registration and tracking database for patients with critical limb ischemia (CLI) who are treated by vascular surgeons. The objective of this project is to elucidate the current status of the medical practice for CLI patients to contribute to the improvement of the quality of medical care. This database, called JAPAN Critical Limb Ischemia Database (JCLIMB), is created on the National Clinical Database (NCD) and collects data of patients’ background, therapeutic measures, early results, and long-term prognosis as long as 5 years after the initial treatment. The limbs managed conservatively are also registered in JCLIMB, together with those treated with surgery and/or endovascular treatment (EVT). In 2019, 1070 CLI limbs (male: 725 limbs, 68%) were registered by 83 facilities. Arteriosclerosis obliterans (ASO) accounted for 98% of the pathogenesis of these limbs. In this manuscript, the background data and the early prognosis of the registered limbs are reported. Although the registration format for the simultaneous surgery of bilateral limbs in NCD was changed to one patient and two limbs, JCLIMB still counted two patients and two limbs to eliminate discrepancy with the past annual reports. (This is a translation of Jpn J Vasc Surg 2022; 31: 157–185.)

## 1. Introduction

Recently, an increasing number of patients with critical limb ischemia (CLI) have undergone medical care at clinical practice sites. Improving the treatment outcome for these patients is an important and urgent issue. Since 2013, the Japanese Society for Vascular Surgery (JSVS) has initiated the project of a nationwide CLI registration and tracking database to obtain CLI epidemiological data that can be shared among the medical staff. The background of CLI limbs, treatment contents, early outcome, and long-term outcome until 5 years after surgery, including non-surgical limbs, are registered in this database. The database was named JAPAN Critical Limb Ischemia Database (JCLIMB) and established on the National Clinical Database (NCD). The primary objective of the JCLIMB project is to elucidate the current status of CLI treatment performed by vascular surgeons in Japan and inform physicians at practice sites of those, thus improving the quality of medical care. The initial registration data, and their tracking data 1 month after registration in 2013–2018, have already been published.^1–6)^ This article reports the basic data registered in 2019.

## 2. JCLIMB

Registration details, including the definition of CLI, have already been described in the 2013 annual report.^1)^ The CLI to be registered was defined according to TASC II^7)^: chronic ischemic rest pain, ulcer, or gangrene attributable to objectively proven arterial occlusive disease. The CLI diagnosis should be confirmed by ankle pressure (AP) below 50 mmHg or toe pressure (TP) below 30 mmHg in limbs with rest pain and by AP below 70 mmHg or TP below 50 mmHg in limbs with ulcer or gangrene.

The same limb can be registered in JCLIMB only once within a 5-year tracking period. When the registered limb is treated at different times or at different institutions, such data should be added only to the tracking items of each limb in JCLIMB, avoiding registration overlap. However, details of the procedure are registered each time in the NCD apart from the registration in JCLIMB. On the other hand, the patient with bilateral CLI can be registered twice for each limb. Based on the NCD regulations, fixing of JCLIMB is done as follows:

Initial registration data: Early April in the following year, tracking data early after treatment (1 month)/6 months after treatment: end of December in the following year, tracking data 1 year after treatment: end of December after 2 years.

Tracking data 2, 3, 4, and 5 years after treatment were registered until the end of December after 3, 4, 5, and 6 years, respectively.

As a general rule, the timing of tracking data registration is accepted within a ±2-month range until 12 months after treatment and within a ±3-month range thereafter. Although the day for tracking data fixing is specified, it is made flexible because in some limbs, follow-up data might be revealed later.

It is very difficult to require facilities participating in NCD to register CLI data because a significant number of registration items in JCLIMB would put too much burden on them. Thus, facilities wishing to participate were recruited. In total, 83 facilities, which registered CLI limbs in 2019 at the time of compiling in December 2021, are listed in the appendix.

Because JCLIMB is considered a registry study on NCD, patient consent to participate in the study and the ethical review of the study at the time of participation in NCD were adopted.

## 3. Comments on the Aggregated Data in 2019

The initial registration data in 2019 were fixed in early April 2020, and the tracking data early after treatment (1 month) were fixed in April 2021. In December 2021, 1070 limbs, belonging to 725 males (68%) and 345 females (32%), were registered in 83 facilities. All data and extracted data on arteriosclerosis obliterans (ASO) were collected according to the registered items. Because ASO accounted for 98% of all limbs, the overall and ASO data exhibited similar tendencies. In the comments, ASO data were presented in parentheses. In addition, because the WIfI classification of the Society for Vascular Surgery (SVS) was reported in 2014 (**Tables 1-1-1–1-1-3****)**,^8)^ JCLIMB made several changes and additions to the registered items, making the WIfI classification possible since 2015 (**Tables 1-2-1–1-2-3**). The total figure was not always consistent, mostly due to missing values, and an explanation for each inconsistency was added. Although the registration format for bilateral simultaneous surgery was changed from two limbs in two patients to two limbs in one patient in the NCD in July 2019, the data was calculated on a limb basis as before in the 2019 JCLIMB annual report to eliminate discrepancy with the past reports.

**Table:** Table 1-1 SVS WIfI classification original^14）^ Table 1-1-1 Wound

Grade	Ulcer	Gangrene
0	No ulcer	No gangrene
	Clinical description: ischemic rest pain (requires typical symptoms+ischemia grade 3); no wound.	
1	Small, shallow ulcer(s) on distal leg or foot; no exposed bone, unless limited to distal phalanx	No gangrene
	Clinical description: minor tissue loss. Salvageable with simple digital amputation (1 or 2 digits) or skin coverage.	
2	Deeper ulcer with exposed bone, joint or tendon; generally not involving the heel; gangrenous changes limited to digits shallow heel ulcer, without calcaneal involvement	Gangrenous changes limited to digits
	Clinical description: major tissue loss salvageable with multiple (3) digital amputations or standard TMA±skin coverage.	
3	Extensive, deep ulcer involving forefoot and/or midfoot; deep, full thickness heel ulcer±calcaneal involvement	Extensive gangrene involving forefoot and/or midfoot; full thickness heel necrosis 6 calcaneal involvement
	Clinical description: extensive tissue loss salvageable only with a complex foot reconstruction or nontraditional TMA (Chopart or Lisfranc); flap coverage or complex wound management needed for large soft tissue defect	

TMA: transmetatarsal amputation

**Table:** Table 1-1-2 Ischemia

Grade	ABI	AP (mmHg)	TP, TcPO_2_ (mmHg)
0	≥0.80	>100	≥60
1	0.60–0.79	70–100	40–59
2	0.40–0.59	50–70	30–39
3	≤0.39	<50	<30

ABI: ankle brachial (pressure) index; PVR: pulse volume recording; SPP: skin perfusion pressure; TP: toe pressure; TcPO_2_: transcutaneous oximetry Patients with diabetes should have TP measurements. If arterial calcification precludes reliable ABI or TP measurements, ischemia should be documented by TcPO_2_, SPP, PVR. If TP and ABI measurements result or in different grades, TP will be the primary determinant of ischemia grade. Flat or minimally pulsatile forefoot PVR=grade 3

**Table:** Table 1-1-3 Foot Infection

Grade	Clinical manifestation of infection	IDSA/PEDIS infection severity*
0	No symptoms or signs of infection	Uninfected
1	Infection present, as defined by the presence of at least 2 of the following items: Mild	Mild
	・Local swelling or induration	
	・Erythema >0.5 to 2 cm around the ulcer	
	・Local tenderness or pain	
	・Local warmth	
	・Purulent discharge (thick, opaque to white, or sanguineous secretion)	
	Local infection involving only the skin and the subcutaneous tissue (without involvement of deeper tissues and without systemic signs as described below)	
	Exclude other causes of an inflammatory response of the skin (e.g., trauma, gout, acute Charcot neuro-osteoarthropathy, fracture, thrombosis, venous stasis)	
2	Local infection (as described above) with erythema >2 cm, or involving structures deeper than skin and subcutaneous tissues (e.g., abscess, osteomyelitis, septic arthritis, fasciitis), and no systemic inflammatory response signs (as described below)	Moderate
3	Local infection (as described above) with the signs of SIRS, as manifested by two or more of the following:	Severe
	・Temperature >38°C or <36°C	
	・Heart rate >90 beats/min	
	・Respiratory rate >20 breaths/min or PaCO_2_ <32 mmHg	
	・White blood cell count >12000 or <4000 cu/mm or 10% immature (band) forms	

*SVS adaptation of Infectious Diseases Society of America (IDSA) and International Working Group on the Diabetic Foot (IWGDF) perfusion, extent/sizePaCO_2_: partial pressure of arterial carbon dioxide; SIRS: systemic inflammatory response syndrome An ischemia may complicate and increase the severity of any infection. Systemic infection may sometimes manifest with other clinical findings, such as hypo-tension, confusion, vomiting, or evidence of metabolic disturbances, such as acidosis, severe hyperglycemia, new-onset azotemia.

**Table:** Table 1-2 SVS WIfI classification: Correlation of WIfI and items in JCLIMB Table 1-2-1 Wound

Grade	Rutherford classification	Ulcer		Sites of gangrene
		Depth of ulcer (University of Texas classification: grade)	Sites of ulcer	
0	Class 4		No ulcer	No gangrene
1	Class 5, 6	I	Any portion	No gangrene
		II, III	Limited to digits	
2	Class 5, 6	I	Heel	Limited to digits
		II, III	Foot: distal metatarsal excluding heel	
3	Class 5, 6	II, III	Foot: proximal metatarsal, heel, ankle, lower leg	Extensive proximal to forefoot

**Table:** Table 1-2-2 Ischemia

Grade	SPP (mmHg; calculating from the formula*)
0	>55
1	42–55
2	35–41
3	<35

* SPP=0.6853×TP+14.48 SPP: skin perfusion pressure; TP: toe pressure

**Table:** Table 1-2-3 Foot Infection

Grade	Local infection: foot	Systemic infection (SIRS)
0	(−)	(−)
1	(+)	(−)
	Involving only the skin and the subcutaneous tissue (Erythema around the ulcer; 0.5–2 cm)	
2	(+)	(−)
	Involving only the skin and the subcutaneous tissue (Erythema around the ulcer; >2 cm), or involving structures deeper than skin and subcutaneous tissues (e.g., abscess, osteomyelitis, septic arthritis, fasciitis)	
3	(+)	(+)

### (1) Pretreatment patients’ background

The pretreatment patients’ background is presented in **Tables 2-1–2-6**. Good blood pressure control was defined as blood pressure below 140/90 mmHg in the absence of diabetes and renal failure or blood pressure below 130/80 mmHg in the presence of these diseases. Good diabetes control was defined as hemoglobin A1c below 7.0% (National Glycohemoglobin Standardization Program [NGSP] value). Good dyslipidemia control was defined as low-density lipoprotein below 100 and 80 mg/dL in the absence and presence of other arteriosclerotic diseases, respectively. The presence of heart failure was judged clinically. The patient was regarded as having heart failure based on a past history of admission due to heart failure, clinical symptoms of heart failure, diagnosis of heart failure confirmed via echocardiography, or reduced cardiac function as revealed by echocardiography even with no clinical heart failure symptoms. Renal dysfunction was graded according to the new chronic kidney disease severity classification of the “Clinical Practice Guideline for the Evaluation and Management of Chronic Kidney Disease 2012”^9)^: Renal dysfunction was absent when the estimated glomerular filtration rate (eGFR) (mL/min/1.73 m^2^) was 60 or higher, and it was graded as G3a, G3b, G4, and G5 when the eGFR was 45–59, 30–44, 15–29, and below 15, respectively. eGFR below 15 in hemodialysis patients was graded as G5D.

**Table:** Table 2 Patients’ background Table 2-1 Patients’ background 1

a. Total														
	n	Sex		Laterality		BMI (Median)	Pathogenesis				Age at registration			
		Male	Female	Right	Left		ASO	TAO	Vasculitis	Others	ASO	TAO	Vasculitis	Others
											Mean (±SD)	Mean (±SD)	Mean (±SD)	Mean (±SD)
Rutherford 4	204	134	70	96	108	21.1	203	0	1	0	74.7 (11.0)	—	74.0 (—)	—
Rutherford 5	709	482	227	395	314	21.3	691	7	6	5	75.1 (9.9)	62.3 (18.3)	72.0 (3.2)	70.0 (9.8)
Rutherford 6	157	109	48	71	86	20.8	153	0	2	2	73.9 (10.9)	—	78.5 (12.0)	70.5 (26.2)
Total	1070	725	345	562	508	21.2	1047	7	9	7	74.9 (10.3)	62.3 (18.3)	73.7 (5.7)	70.1 (13.3)
b. ASO														
	n	Sex		Laterality		BMI (Median)	Age at registration							
		Male	Female	Right	Left		Mean (±SD)							
Rutherford 4	203	134	69	96	107	21.1	74.7 (11.0)							
Rutherford 5	691	473	218	387	304	21.3	75.1 (9.9)							
Rutherford 6	153	107	46	69	84	21.0	73.9 (10.9)							
Total	1047	714	333	552	495	21.2	74.9 (10.3)							

Vasculitis: Takayasu’s arteritis, Collagen disease, Behcet disease, FMD etc., excluding TAOOthers: others including debranch bypasses for TEVAR or EVARASO: arteriosclerosis obliterans; TAO: thromboangiitis obliterans; FMD: fibromuscular dysplasia; BMI: body mass index;TEVAR: thoracic endovascular aortic aneurysm repair; EVAR: endovascular aortic aneurysm repairSimultaneous bilateral treatments in one case were counted as 2 limbs in 2 cases.

**Table:** Table 2-2 Patients’ background 2

a. Total															
	Diabetes			Diabetes therapy			Hypertension			Dyslipidemia			Smoking		
	(−)	(+)		Diet therapy	Medication	Insulin therapy	(−)	(+)		(−)	(+)		(−)	(+)	
		Management						Management			Management			Ex-smoker	Current smoker
		Good	Poor					Good	Poor		Good	Poor			
Rutherford 4	91	87	26	21	63	29	60	132	12	132	67	5	104	67	33
Rutherford 5	237	349	123	75	221	176	172	458	79	403	273	33	281	334	94
Rutherford 6	50	68	39	18	34	55	45	89	23	92	56	9	60	73	24
Total	378	504	188	114	318	260	277	679	114	627	396	47	445	474	151
b. ASO															
	Diabetes			Diabetes therapy			Hypertension			Dyslipidemia			Smoking		
	(−)	(+)		Diet therapy	Medication	Insulin therapy	(−)	(+)		(−)	(+)		(−)	(+)	
		Management						Management			Management			Ex-smoker	Current smoker
		Good	Poor					Good	Poor		Good	Poor			
Rutherford 4	90	87	26	21	63	29	60	131	12	131	67	5	103	67	33
Rutherford 5	223	346	122	74	218	176	168	445	78	391	269	31	274	325	92
Rutherford 6	46	68	39	18	34	55	43	87	23	90	54	9	58	72	23
Total	359	501	187	113	315	260	271	663	113	612	390	45	435	464	148

HbA1c: hemoglobin A1c; LDL: low-density lipoprotein; NGSP: national glycohemoglobin standardization programBlood pressure management good: diabetes or renal failure (−) <140/90 mmHg (+) <130/80 mmHg. Diabetes management good: HbA1c<7.0% (NGSP). Dyslipidemia management good: other sclerotic lesions (−) LDL<100 mg/dL, (+) LDL<80 mg/dL.

**Table:** Table 2-3 Patients’ background 3

a. Total														
	Ischemic heart disease				Heart failure		Cerebrovascular disease		Renal dysfunction					
	(−)	(+)			(−)	(+)	(−)	(+)	(−)	(+)				
		Medical treatment	PCI	CABG						G3a	G3b	G4	G5	G5D
Rutherford 4	156	17	19	12	179	25	170	34	94	13	19	7	1	70
Rutherford 5	403	79	132	95	610	99	561	148	223	77	60	38	4	307
Rutherford 6	84	23	29	21	118	39	130	27	56	14	13	5	3	66
Total	643	119	180	128	907	163	861	209	373	104	92	50	8	443
b. ASO														
	Ischemic heart disease				Heart failure		Cerebrovascular disease		Renal dysfunction					
	(−)	(+)			(−)	(+)	(−)	(+)	(−)	(+)				
		Medical treatment	PCI	CABG						G3a	G3b	G4	G5	G5D
Rutherford 4	155	17	19	12	178	25	169	34	93	13	19	7	1	70
Rutherford 5	386	79	131	95	596	95	544	147	212	75	56	37	4	307
Rutherford 6	81	23	28	21	115	38	126	27	53	14	13	5	3	65
Total	622	119	178	128	889	158	839	208	358	102	88	49	8	442

PCI: percutaneous coronary intervention; CABG: coronary arterial bypass grafting　Heart failure (+): history of admission due to heart failure, clinical symptoms due to heart failure confirmed by ultrasound examination, apparently decreased cardiac function by ultrasound examination without clinical symptoms Renal dysfunction: (−) (60≦), G3a (45～59), G3b (30～44), G4 (15～29), G5 (<15), G5D (<15 with hemodialysis). New CKD risk stratification by eGFR (mL/min/1.73 m^2^) in “Clinical Practice Guidebook for Diagnosis and Treatment of Chronic Kidney Disease 2012”　eGFR: estimated glomerular filtration rate; CKD: chronic kidney disease

**Table:** Table 2-4 Patients’ background 4

a. Total															
	Malignant neoplasm				Site of malignant neoplasm										
	(−)	(+)			Head and neck	Esophagus	Lung	Stomach	Hepatobiliary pancreas	Colon	Breast	Uterus	Ovarium	Prostate	Others
		History of cancer	Under treatment*	Unknown											
Rutherford 4	179	16	8	1	2	2	4	5	2	6	1	0	0	0	4
Rutherford 5	635	47	21	6	3	0	11	19	5	14	3	3	0	7	13
Rutherford 6	139	11	6	1	2	2	2	5	1	2	1	0	0	1	3
Total	953	74	35	8	7	4	17	29	8	22	5	3	0	8	20
b. ASO															
	Malignant neoplasm				Site of malignant neoplasm										
	(−)	(+)			Head and neck	Esophagus	Lung	Stomach	Hepatobiliary pancreas	Colon	Breast	Uterus	Ovarium	Prostate	Others
		History of cancer	Under treatment*	Unknown											
Rutherford 4	178	16	8	1	2	2	4	5	2	6	1	0	0	0	4
Rutherford 5	620	45	20	6	3	0	10	18	5	13	3	3	0	7	13
Rutherford 6	136	11	6	0	2	2	2	5	1	2	1	0	0	1	3
Total	934	72	34	7	7	4	16	28	8	21	5	3	0	8	20

*Including palliative therapy or recurrence

**Table:** Table 2-5 Patients’ background 5

a. Total													
	Contralateral limb occlusive lesions							Vascular lesions excluding occlusion					
	(−)	(+)						(−)	TAA	AAA (including IAA)	Peripheral artery aneurysm	Carotid stenosis	Others
		Asymptomatic	Intermittent claudication	CLI			Post-treatment						
				R4	R5	R6							
Rutherford 4	65	44	16	29	0	0	41	186	2	7	0	5	4
Rutherford 5	187	212	37	16	117	3	125	652	6	16	0	21	14
Rutherford 6	34	53	4	1	12	19	29	136	0	1	2	13	5
Total	286	309	57	46	129	22	195	974	8	24	2	39	23
b. ASO													
	Contralateral limb occlusive lesions							Vascular lesions excluding occlusion					
	(−)	(+)						(−)	TAA	AAA (including IAA)	Peripheral artery aneurysm	Carotid stenosis	Others
		Asymptomatic	Intermittent claudication	CLI			Post-treatment						
				R4	R5	R6							
Rutherford 4	65	44	16	29	0	0	40	185	2	7	0	5	4
Rutherford 5	176	209	36	15	116	3	124	637	5	15	0	21	13
Rutherford 6	31	52	4	1	12	19	29	134	0	1	0	13	5
Total	272	305	56	45	128	22	193	956	7	23	0	39	22

CLI: critical limb ischemia; TAA: thoracic aortic aneurysm; AAA: abdominal aortic aneurysm; IAA: iliac artery aneurysm

**Table:** Table 2-6 Patients’ background 6

a. Total								
	Fatty acid							
	Arachidonic acid (AA)		Eicosapentaenoic acid (EPA)		Docosahexaenoic acid (DHA)		EPA/AA	
	n	Median	n	Median	n	Median	n	Median
Rutherford 4	5	201.6	5	85.4	5	154.5	5	0.4
Rutherford 5	12	188.2	12	48.2	12	100.9	12	0.3
Rutherford 6	3	66.8	3	26.3	3	58.5	3	0.4
Total	20	193.7	20	53.7	20	100.9	20	0.3
b. ASO								
	Fatty acid							
	Arachidonic acid (AA)		Eicosapentaenoic acid (EPA)		Docosahexaenoic acid (DHA)		EPA/AA	
	n	Median	n	Median	n	Median	n	Median
Rutherford 4	5	201.6	5	85.4	5	154.5	5	0.4
Rutherford 5	12	188.2	12	48.2	12	100.9	12	0.3
Rutherford 6	3	66.8	3	26.3	3	58.5	3	0.4
Total	20	193.7	20	53.7	20	100.9	20	0.3

The causes of the arterial occlusion of the limb were ASO in 1047 (98%) limbs, thromboangiitis obliterans (TAO) in 7, vasculitis (Takayasu’s arteritis, collagen disease, Behçet’s disease, and fibromuscular dysplasia excluding TAO) in 9, and others in 7. Comorbidities consisted of diabetes in 65% (66%) of the limbs, hypertension in 74% (74%), dyslipidemia in 41% (42%), ischemic heart disease in 40% (41%), heart failure in 15% (15%), cerebrovascular disease in 20% (20%), dialysis for renal failure in 41% (42%), past medical history of malignant neoplasm or that being treated in 11% (11%), arterial occlusive lesions in the contralateral limb in 73% (74%), and smoking (ex- and current) in 58% (58%).

The problems and considerations on these spreadsheets are described below. In **Table 2-4**, the total number of malignant neoplasm sites is larger than that of malignant neoplasms. This is because multiple selections of malignant neoplasm sites were possible. The blood flow data (ankle brachial index, toe brachial index [TBI], and skin perfusion pressure [SPP]) of contralateral limbs were omitted in this report as there were many missing values, though they had been displayed until 2018.

### (2) Conditions of limb ischemia

Limb ischemia pretreatment conditions are presented in **Tables 3-1** to **3-6**. Regarding the walking function (Taylor classification),^10)^ patients who could walk outdoors or indoors independently, including with a cane, were regarded as “ambulatory,” whereas those unable to walk but able to stand on their own legs during transfer from the bed to a wheel chair were designated as “ambulatory/homebound.”

**Table:** Table 3 Pretreatment condition Table 3-1 Pretreatment condition 1

a. Total																										
	Ambulatory function (Taylor’s classification)			Site of ulcer							Depth of ulcer (University of Texas classification: grade)			Site of gangrene							Main site of ulcer/gangrene to be treated					
	Ambulatory	Ambulatory/homebound	Non-ambulatory	Digits	Foot distal metatarsal	Foot proximal metatarsal	Heel	Ankle	Lower leg	Only gangrene w/o ulcer	I	II	III	Digits	Foot distal metatarsal	Foot proximal metatarsal	Heel	Ankle	Lower leg	Only ulcer w/o gangrene	Digits	Foot distal metatarsal	Foot proximal metatarsal	Heel	Ankle	Lower leg
Rutherford 4	148	28	28																							
Rutherford 5	431	143	135	505	88	19	57	17	9	74	403	112	127	332	49	13	27	4	3	303	544	86	14	42	15	6
Rutherford 6	58	40	59	44	44	24	46	10	14	26	29	37	68	51	47	28	42	10	10	21	33	39	18	46	9	12
Total	637	211	222	549	132	43	103	27	23	100	432	149	195	383	96	41	69	14	13	324	577	125	32	88	24	18
b. ASO																										
	Ambulatory function (Taylor’s classification)			Site of ulcer							Depth of ulcer (University of Texas classification: grade)			Site of gangrene							Main site of ulcer/gangrene to be treated					
	Ambulatory	Ambulatory/homebound	Non-ambulatory	Digits	Foot distal metatarsal	Foot proximal metatarsal	Heel	Ankle	Lower leg	Only gangrene w/o ulcer	I	II	III	Digits	Foot distal metatarsal	Foot proximal metatarsal	Heel	Ankle	Lower leg	Only ulcer w/o gangrene	Digits	Foot distal metatarsal	Foot proximal metatarsal	Heel	Ankle	Lower leg
Rutherford 4	147	28	28																							
Rutherford 5	417	140	134	492	85	18	57	17	9	73	394	108	123	326	48	11	27	4	3	294	530	84	12	42	15	6
Rutherford 6	56	39	58	44	43	23	46	10	13	25	28	36	67	48	46	26	41	10	10	21	32	38	18	45	9	11
Total	620	207	220	536	128	41	103	27	22	98	422	144	190	374	94	37	68	14	13	315	562	122	30	87	24	17

University of Texas classification: grade (I: superficial, not involving tendon, capsule, or bone, II: penetrating to tendon/capsule, III: penetrating to bone or joint)

**Table:** Table 3-2 Pretreatment condition 2

a. Total																								
	Temperature >38℃		Blood test								Hemodynamics								Infection*^1)^					
	(−)	(+)	WBC		CRP		Alb		Cr		ABI		TBI		SPP		Toe pressure		Local (foot)				Systemic	
			n	Median	n	Median	n	Median	n	Median	n	Median	n	Median	n	Median	n	Median	Uninfected	Skin or subcutaneous tissue (erythema)*^2)^		Deep tissue*^3)^	SIRS*^4)^	
																				≦2 cm	>2 cm		(+)	(−)
Rutherford 4	200	4	201	6500	196	0.52	193	3.6	200	1.35	109	0.57	6	0.55	85	19.0	7	76.0	24	1	1	1	3	199
Rutherford 5	694	15	698	7130	676	1.13	673	3.4	695	1.64	450	0.62	33	0.29	440	21.0	36	35.0	444	168	41	54	9	700
Rutherford 6	131	26	153	8800	151	4.06	150	2.9	152	1.40	88	0.68	4	0.21	79	24.0	5	33.0	45	30	26	56	13	144
Total	1025	45	1052	7235	1023	1.19	1016	3.3	1047	1.53	647	0.62	43	0.31	604	21.0	48	36.5	513	199	68	111	25	1043
b. ASO																								
	Temperature >38℃		Blood test								Hemodynamics								Infection*^1)^					
	(−)	(+)	WBC		CRP		Alb		Cr		ABI		TBI		SPP		Toe pressure		Local (foot)				Systemic	
			n	Median	n	Median	n	Median	n	Median	n	Median	n	Median	n	Median	n	Median	Uninfected	Skin or subcutaneous tissue (erythema)*^2)^		Deep tissue*^3)^	SIRS*^4)^	
																							(+)	(−)
																				≦2 cm	>2 cm			
Rutherford 4	199	4	200	6500	195	0.52	192	3.6	199	1.36	108	0.57	6	0.55	84	19.0	7	76.0	23	1	1	1	3	198
Rutherford 5	676	15	681	7100	659	1.13	656	3.4	678	1.71	441	0.62	33	0.29	429	21.0	36	35.0	433	163	39	54	9	682
Rutherford 6	127	26	150	8750	148	4.00	148	2.9	150	1.42	88	0.68	4	0.21	78	24.0	5	33.0	45	29	25	54	13	140
Total	1002	45	1031	7200	1002	1.19	996	3.3	1027	1.57	637	0.62	43	0.31	591	21.0	48	36.5	501	193	65	109	25	1020

WBC: white blood cell; CRP: C reactive protein; Alb: albumin; Cr: creatinine; ABI: ankle brachial (pressure) index; TBI: toe brachial (pressure) index; SPP: skin perfusion pressure; SIRS: systemic inflammatory response syndrome　*^1)^ Presence of infection was defined as by the presence of at least 2 of the following issues: ①Local swelling or induration, ②erythema >5 mm to ≦2 cm around the ulcer, ③Local tenderness or pain, ④Local warmth, ⑤Purulent discharge (thick opaque to white, or sanguineous secretion) *^2)^ Local infection are skin and subcutaneous tissue was classified by the spreading of erythema (≦2 cm or >2 cm around the ulcer/gangrene) *^3)^ Local infection involving structures deeper than skin and subcutaneous tissues (e.g., abscess, osteomyelitis, septic arthritis, fasciitis) *^4)^ The signs of SIRS are manifested by two or more of the following: ①Temperature >38°C or <36°C, ②Heart rate >90 beats/min, ③Respiratory rate >20  breaths/min or PaCO_2_<32 mmHg, ④White blood cell count >12000 or <4000 cu/mm or 10% immature (band) forms

**Table:** Table 3-3 Pretreatment condition 3

a. Total																
	Diagnostic imaging			Site of occlusion			TASC II classification aortoiliac					TASC II classification femoropopliteal				
	IADSA	CTA	Others	Aortoiliac	Femoropop	Lower leg/foot	A	B	C	D	No lesion	A	B	C	D	No lesion
Rutherford 4	111	116	13	55	137	89	11	14	7	13	4	6	20	28	93	14
Rutherford 5	435	410	19	144	423	457	50	23	18	36	9	86	97	89	246	105
Rutherford 6	101	91	3	36	84	105	13	3	3	17	0	21	14	17	54	28
Total	647	617	35	235	644	651	74	40	28	66	13	113	131	134	393	147
b. ASO																
	Diagnostic imaging			Site of occlusion			TASC II classification aortoiliac					TASC II classification femoropopliteal				
	IADSA	CTA	Others	Aortoiliac	Femoropop	Lower leg/foot	A	B	C	D	No lesion	A	B	C	D	No lesion
Rutherford 4	110	116	13	55	137	88	11	14	7	13	4	6	20	28	93	13
Rutherford 5	427	397	18	139	415	445	46	23	18	35	9	86	96	87	241	100
Rutherford 6	99	88	3	35	82	102	13	3	3	16	0	21	14	17	53	26
Total	636	601	34	229	634	635	70	40	28	64	13	113	130	132	387	139

IADSA: intra-arterial digital subtraction angiography; CTA: computed tomography angiography

**Table:** Table 3-4 Pretreatment condition 4

a. Total														
	Bollinger Score													
	Common femoral		Deep femoral		Superficial femoral: proximal		Superficial femoral: distal		Popliteal: proximal		Popliteal: distal		Tibioperoneal trunk	
	n	Median	n	Median	n	Median	n	Median	n	Median	n	Median	n	Median
Rutherford 4	62	1.0	62	1.0	62	6.0	62	6.0	61	4.0	61	3.0	61	3.0
Rutherford 5	312	1.0	313	1.0	310	3.0	310	4.0	312	3.0	312	2.0	309	3.0
Rutherford 6	79	2.0	79	1.0	79	4.0	79	4.0	79	3.0	80	2.0	80	4.0
Total	453	1.0	454	1.0	451	4.0	451	5.0	452	3.0	453	2.0	450	3.0
b. ASO														
	Bollinger Score													
	Common femoral		Deep femoral		Superficial femoral: proximal		Superficial femoral: distal		Popliteal: proximal		Popliteal: distal		Tibioperoneal trunk	
	n	Median	n	Median	n	Median	n	Median	n	Median	n	Median	n	Median
Rutherford 4	62	1.0	62	1.0	62	6.0	62	6.0	61	4.0	61	3.0	61	3.0
Rutherford 5	309	1.0	310	1.0	307	3.0	307	4.0	309	3.0	309	2.0	306	3.0
Rutherford 6	78	2.0	78	1.0	78	3.5	78	4.0	78	3.0	79	2.0	79	4.0
Total	449	1.0	450	1.0	447	4.0	447	5.0	448	3.0	449	2.0	446	3.0

**Table:** Table 3-5 Pretreatment condition 5

a. Total														
	Bollinger Score													
	Posterior tibial: proximal		Posterior tibial: distal		Anterior tibial: proximal		Anterior tibial: distal		Peroneal: proximal		Peroneal: distal		Foot	
	n	Median	n	Median	n	Median	n	Median	n	Median	n	Median	n	Median
Rutherford 4	61	15.0	61	13.0	60	5.0	59	6.0	58	4.0	58	4.0	49	3.0
Rutherford 5	306	13.0	305	13.0	308	13.0	306	13.0	307	6.0	306	6.0	281	6.0
Rutherford 6	80	15.0	79	13.0	80	11.5	79	6.0	80	6.0	79	6.0	67	13.0
Total	447	13.0	445	13.0	448	13.0	444	13.0	445	6.0	443	6.0	397	6.0
b. ASO														
	Bollinger Score													
	Posterior tibial: proximal		Posterior tibial: distal		Anterior tibial: proximal		Anterior tibial: distal		Peroneal: proximal		Peroneal: distal		Foot	
	n	Median	n	Median	n	Median	n	Median	n	Median	n	Median	n	Median
Rutherford 4	61	15.0	61	13.0	60	5.0	59	6.0	58	4.0	58	4.0	49	3.0
Rutherford 5	304	13.0	303	13.0	305	13.0	303	13.0	305	6.0	303	6.0	278	6.0
Rutherford 6	79	15.0	78	13.0	79	12.0	78	6.0	79	6.0	78	6.0	66	13.0
Total	444	13.0	442	13.0	444	13.0	440	13.0	442	6.0	439	6.0	393	6.0

**Table:** Table 3-6 SVS WIfI classification

a. Total																
	Wound				Ischemia				foot Infection				Stage			
	0	1	2	3	0	1	2	3	0	1	2	3	1	2	3	4
Rutherford 4	204	0	0	0	14	30	17	83	24	1	1	1	4	17	1	1
Rutherford 5	0	236	317	144	55	84	70	382	444	165	93	5	46	42	185	310
Rutherford 6	0	5	25	115	13	14	10	72	45	27	72	13	2	2	8	91
Total	204	241	342	259	82	128	97	537	513	193	166	19	52	61	194	402
b. ASO																
	Wound				Ischemia				foot Infection				Stage			
	0	1	2	3	0	1	2	3	0	1	2	3	1	2	3	4
Rutherford 4	203	0	0	0	14	29	17	83	23	1	1	1	3	17	1	1
Rutherford 5	0	228	311	140	54	84	68	372	433	160	91	5	46	41	180	303
Rutherford 6	0	5	25	111	13	14	10	71	45	26	69	13	2	2	8	90
Total	203	233	336	251	81	127	95	526	501	187	161	19	51	60	189	394

Regarding the state of local tissue defect (Texas University Classification),^11)^ the most severe lesion, the main treatment target, was evaluated. SPP was measured on the foot (base of the toe, dorsum of the foot, or sole), and a lower value was used. To perform the WIfI classification, the ulcer and gangrene sites were separately registered. Although SPP is widely used as an objective index for evaluating ischemia in Japan, the ischemic grading criteria using SPP is not shown in the WIfI classification, in which TP is given top priority. Therefore, in JCLIMB, the SPP value was converted to TP using the conversion equation SPP=0.6853× TP +14.48 from the correlation data of SPP and TP reported in Japan^12)^ and applied for WIfI ischemic grading (**Table 1-2-2**). Because the ischemic grade of the WIfI classification based on the SPP value was newly defined in the Japanese PAD guideline revised in 2022, the classification method will be changed in the annual report after 2020.

The lesion was considered infected when it showed two or more of the following symptoms: local swelling or induration, erythema >0.5 cm around the ulcer, local tenderness or pain, local warmth, and purulent discharge (thick, opaque to white, or sanguineous secretion). In addition, local infections involving only the skin and the subcutaneous tissue, and those involving structures deeper than the skin and subcutaneous tissues, were separately registered. Local infections involving only the skin and the subcutaneous tissue were differentiated based on the size of the erythema around the ulcer, ≦2 or >2 cm. Systemic inflammatory response syndrome, indicating systemic infection, was manifested by two or more of the following symptoms: temperature >38°C or <36°C, heart rate >90 beats/min, respiratory rate >20 breaths/min or PaCO_2_<32 mmHg, white blood cell count >12,000 or <4,000 cu/mm, or 10% immature (band) forms. The arteries in the ankle joint region were classified as foot arteries.

In the pretreatment, 60% (59%) of the patients were ambulatory, 20% (20%) were ambulatory/homebound, and 21% (21%) were non-ambulatory. On the Rutherford classification (R),^13)^ limbs with categories R4, R5, and R6 accounted for 19% (19%), 66% (66%), and 15% (15%) of the limbs, respectively. The median ABI, TBI, and SPP of the measured limbs were 0.62 (0.62), 0.31 (0.31), and 21 mmHg (21 mmHg), respectively. The occlusive legion was located in the aortoiliac artery in 22% (22%) of the limbs, the femoropopliteal artery in 60% (61%) of the limbs, and the crural or foot artery in 61% (61%) of the limbs. The multiple occlusive lesions were located in the aortoiliac and femoropopliteal arteries in 12% (12%) of limbs, the aortoiliac artery and the crural or foot artery in 6% (6%), the femoropopliteal artery and the crural or foot artery in 30% (30%), and the aortoiliac artery and the femoropopliteal artery and the crural or foot artery in 5% (5%).

We were able to apply the WIfI classification with sufficient data to 709 limbs (694 limbs). On the WIfI classification, limbs with the stages 1, 2, 3, and 4 accounted for 7% (7%), 9% (9%), 27% (27%), and 57% (57%) of the limbs, respectively.

The problems and considerations on these spreadsheets are described below. In **Table 3-1**, the total number of ambulatory function differed from the total number of the main sites of ulcer/gangrene to be treated. This is because there were missing values in the main sites of ulcer/gangrene to be treated. In **Table 3-3**, the total number of limbs in the TASC II classification differed from the number in each column of the site of occlusion. In the “aortoiliac” lesion, a decreased number of that in the TASC II classification may have been due to input omission. In the “femoropopliteal” lesion, an increased number of that in TASC II may have been due to the crural lesions. In **Table 3-6**, there was some dissociation between the R and Wound grades. This may be due to the R grade’s obscure definition. For example, extensive gangrene involving the forefoot is classified in R5 and W3, whereas a shallow ulcer without exposure of the distal leg bone is classified in R6 and W1. In **Table 3-6**, 82 limbs (81 limbs) were registered as ischemic grade 0 in the WIfI classification. By definition, a limb with ischemic grade 0 has an ABI 0.8 or higher or AP higher than 100 mmHg, or if arterial calcification precludes reliable ABI or AP measurements, TP of 60 mmHg or higher or TcPO_2_ 60 mmHg or higher (SPP 55 mmHg or higher in JCLIMB) (**Table 1-1-2**). There should be no limb with ischemic grade 0 because the CLI registered in JCLIMB is defined according to TASC II. The limbs might be clinically judged to be CLI irrespective of the objective ischemic index, although details are unknown. **Table 3-6** demonstrates that there were three limbs (3 limbs) in which infection was confirmed in R4 limbs, despite the absence of a local wound by definition of R4. This may occur because tissue loss is not always requisite for fI grade. In **Table 3-6**, the numbers of wound, ischemia, foot infection, and stage are different. This is because there were missing values in items required for grading.

### (3) Treatment

**Tables 4-1** to **4-6** present the CLI treatment data. Revascularizations of the affected limbs were performed in 94% (95%) of the registered limbs, and primary major amputations were performed in 2.3% (2.4%) of the registered limbs. Among the surgical reconstruction procedures, distal bypass accounted for 54% (54%). Endovascular treatment (EVT), including EVT alone and hybrid treatment with surgical reconstruction, accounted for 54% (55%) of the total revascularization procedures. The EVT applied to the crural or foot artery accounted for 37% (36%) of the total EVT.

**Table:** Table 4 Treatment Table 4-1 Treatment 1

a. Total																				
	Treatment					Angiogenic therapy			Amputation							Reoperation				
	Pharmacological therapy	Angiogenic therapy	Arterial reconstruction	Major amputation	Lumber sympathectomy	Bone marrow	Peripheral blood	Others	Toe	Metatarsal	Chopart/Lisfranc	Syme	Below knee	Above knee-knee disarticulation	Hip disarticulation	Unknown	(−)	(+)		
																		1X	2X	3X≦
Rutherford 4	66	0	189	1	0	0	0	0	0	0	0	0	0	0	0	1	141	33	14	15
Rutherford 5	212	1	675	10	0	0	0	1	14	9	0	0	0	0	0	9	542	86	28	44
Rutherford 6	45	1	147	14	0	0	1	0	1	3	0	0	2	1	0	4	130	16	6	1
Total	323	2	1011	25	0	0	1	1	15	12	0	0	2	1	0	14	813	135	48	60
b. ASO																				
	Treatment					Angiogenic therapy			Amputation							Reoperation				
	Pharmacological therapy	Angiogenic therapy	Arterial reconstruction	Major amputation	Lumber sympathectomy	Bone marrow	Peripheral blood	Others	Toe	Metatarsal	Chopart/Lisfranc	Syme	Below knee	Above knee-knee disarticulation	Hip disarticulation	Unknown	(−)	(+)		
																		1X	2X	3X≦
Rutherford 4	65	0	188	1	0	0	0	0	0	0	0	0	0	0	0	1	140	33	14	15
Rutherford 5	205	1	659	10	0	0	0	1	14	8	0	0	0	0	0	9	531	82	27	42
Rutherford 6	45	1	143	14	0	0	1	0	1	3	0	0	2	1	0	4	127	15	6	1
Total	315	2	990	25	0	0	1	1	15	11	0	0	2	1	0	14	798	130	47	58

**Table:** Table 4-2 Treatment 2

a. Total															
	Bypass											TEA			EVT
	Aorta-aorta	Aorta (with suprarenal clamp)	Aorta-femoral*	Femora-proximal popliteal	Femoral-distal popliteal	Femoral-crural/foot	Popliteal-crural/foot	Anatomical others	Axillary-femoral	Femoral-femoral	Extraanatomical others	Aorta/iliac	Femoral/popliteal	Others	
Rutherford 4	0	0	6	10	10	20	19	2	2	3	2	3	13	0	112
Rutherford 5	0	0	8	30	40	94	109	3	4	4	4	2	44	6	408
Rutherford 6	0	0	0	7	8	11	24	0	4	6	1	0	11	1	90
Total	0	0	14	47	58	125	152	5	10	13	7	5	68	7	610
b. ASO															
	Bypass											TEA			EVT
	Aorta-aorta	Aorta (with suprarenal clamp)	Aorta-femoral*	Femora-proximal popliteal	Femoral-distal popliteal	Femoral-crural/foot	Popliteal-crural/foot	Anatomical others	Axillary-femoral	Femoral-femoral	Extraanatomical others	Aorta/iliac	Femoral/popliteal	Others	
Rutherford 4	0	0	6	10	10	20	18	2	2	3	2	3	13	0	112
Rutherford 5	0	0	8	30	40	89	107	3	4	4	1	2	44	6	401
Rutherford 6	0	0	0	6	6	11	24	0	4	5	1	0	11	1	90
Total	0	0	14	46	56	120	149	5	10	12	4	5	68	7	603

TEA: thromboendarterectomy; EVT: endovascular treatment/therapy　*Including aorta-femoral, aorta-iliac, ilio-femoral bypass

**Table:** Table 4-3 Treatment 3

a. Total																	
	EVT				Vascular material					Vein usage					Vein quality		
	Aorta/iliac	Femoral/popliteal	Tibioperoneal/foot	Others	Polyester	ePTFE	Vein	Others	(−)	In-situ	Non-reversed	Reversed	Spliced	Patch	Patch	Good	Poor
Rutherford 4	37	61	36	7	4	16	58	0	17	4	17	26	4	9	5	50	3
Rutherford 5	105	200	196	4	11	34	254	3	36	34	109	80	18	20	13	231	10
Rutherford 6	23	51	50	1	2	15	47	1	11	1	21	20	0	5	3	39	5
Total	165	312	282	12	17	65	359	4	64	39	147	126	22	34	21	320	18
b. ASO																	
	EVT				Vascular material					Vein usage					Vein quality		
	Aorta/iliac	Femoral/popliteal	Tibioperoneal/foot	Others	Polyester	ePTFE	Vein	Others	(−)	In-situ	Non-reversed	Reversed	Spliced	Patch	Patch	Good	Poor
Rutherford 4	37	61	36	7	4	16	57	0	17	4	16	26	4	9	5	49	3
Rutherford 5	102	198	192	4	11	34	247	3	34	33	106	79	16	20	13	224	10
Rutherford 6	23	51	50	1	2	14	44	1	11	1	19	19	0	5	3	36	5
Total	162	310	278	12	17	64	348	4	62	38	141	124	20	34	21	309	18

ePTFE: expanded polytetrafluoroethylene

**Table:** Table 4-4 Treatment 4

a. Total																			
	Distal bypass																		
	Proximal anastomosis								Distal anastomosis		Distal anastomosis: site of crural artery				Distal anastomosis: site of foot artery				
	External iliac	Common femoral	Deep femoral	Superficial femoral	Proximal popliteal	Distal popliteal	Crural	Others	Crural	Foot	Tibioperoneal trunk	Posterior tibial	Anterior tibial	Peroneal	Posterior tibial	Anterior tibial	Peroneal	Dorsal pedis	Plantar
Rutherford 4	1	13	2	4	5	5	6	3	23	16	2	14	3	4	3	3	0	9	1
Rutherford 5	0	54	4	34	25	71	7	7	77	126	4	42	24	7	22	17	2	77	12
Rutherford 6	0	6	1	4	8	15	0	1	17	18	2	10	4	1	4	2	0	11	1
Total	1	73	7	42	38	91	13	11	117	160	8	66	31	12	29	22	2	97	14
b. ASO																			
	Distal bypass																		
	Proximal anastomosis								Distal anastomosis		Distal anastomosis: site of crural artery				Distal anastomosis: site of foot artery				
	External iliac	Common femoral	Deep femoral	Superficial femoral	Proximal popliteal	Distal popliteal	Crural	Others	Crural	Foot	Tibioperoneal trunk	Posterior tibial	Anterior tibial	Peroneal	Posterior tibial	Anterior tibial	Peroneal	Dorsal pedis	Plantar
Rutherford 4	1	13	2	4	5	5	5	3	22	16	2	13	3	4	3	3	0	9	1
Rutherford 5	0	53	4	32	23	70	6	7	76	120	4	42	24	6	20	17	1	75	10
Rutherford 6	0	6	1	4	8	15	0	1	17	18	2	10	4	1	4	2	0	11	1
Total	1	72	7	40	36	90	11	11	115	154	8	65	31	11	27	22	1	95	12

**Table:** Table 4-5 Treatment 5

a. Total						
	Pharmacological therapy					
	Antiplatelet	Anticoagulant	Prostaglandin	Heparin	Statin	Others
Rutherford 4	96	15	1	6	14	6
Rutherford 5	289	31	23	32	56	23
Rutherford 6	66	9	7	8	9	3
Total	451	55	31	46	79	32
b. ASO						
	Pharmacological therapy					
	Antiplatelet	Anticoagulant	Prostaglandin	Heparin	Statin	Others
Rutherford 4	95	15	1	6	14	5
Rutherford 5	283	30	23	32	55	20
Rutherford 6	66	9	7	8	9	3
Total	444	54	31	46	78	28

**Table:** Table 4-6 Treatment 6

a. Total				
	Femoro-proximal popliteal bypass	Femoro-distal popliteal bypass	Femoro-crural/foot bypass	Popliteal-crural/foot bypass
Polyester	3	1	0	1
ePTFE	28	7	8	3
Vein	17	49	115	145
Artery	1	0	6	5
Others	1	2	0	0
(−)	0	0	0	0
Total	50	59	129	154
b. ASO				
	Femoro-proximal popliteal bypass	Femoro-distal popliteal bypass	Femoro-crural/foot bypass	Popliteal-crural/foot bypass
Polyester	3	1	0	1
ePTFE	28	7	8	3
Vein	16	47	110	142
Artery	1	0	6	5
Others	1	2	0	0
(−)	0	0	0	0
Total	49	57	124	151

ePTFE: expanded polytetrafluoroethylene

The problems and considerations on these spreadsheets are described below. In **Table 4-1**, the sum of the number of cells in treatment is larger than that of the number of registered limbs, 1070 (1049), because more than one treatment method can be selected. In **Table 4-1**, the discrepancy in the number of major amputation to the number of detail of amputation was caused by “unused.” In the column of “vein usage” of **Table 4-3**, how the autologous veins were used was described when they were selected as vascular conduits. The sum of the number in the column of vein usage, “in-situ,” “non-reversed,” “reversed,” “spliced,” and “patch,” is larger than the sum of the number in the column of vein in vascular prosthesis. It could be because of selecting multiple vein usage for arterial reconstruction in a limb. The sum of the number in the column of vein in vascular prosthesis is identical to the sum of the number in the column of vein quality. Vascular prosthesis (−) included an endarterectomy without a patch angioplasty. In **Table 4-4**, the sum of the number of proximal anastomosis is not equal to the sum of the number of distal anastomosis. This was because multiple arteries could be selected in each anastomosis. The total number of distal anastomosis sites of the foot artery is larger than that of distal anastomosis “foot.” This was because multiple sites were selected in dual bypass.

**Table 4-6** summarizes the vascular grafts used for the infrainguinal arterial reconstruction. For example, the total number of vascular graft in the column of femoral–proximal popliteal artery bypass was 50 (49), which was higher than 47 (46), the number of actual applications in **Table 4-2**. This was because multiple graft materials could be selected when multiple procedures, such as a sequential bypass procedure and TEA, can be performed simultaneously for arterial reconstruction in the lower limb.

### (4) Outcomes early (1 month) after treatment

**Tables 5-1** to **5-8** present the outcomes early (1 month) after treatment. At the time of summary count at the end of April 2021, follow-up data 1 month after treatment were obtained in 886 limbs (83%), including 866 limbs (83%) with ASO. Data were collected according to the severity of the local limb conditions (Rutherford classification) and treatment measures (EVT alone or surgical reconstruction with/without EVT). The mortality rate was 3.6% (3.7%) in the whole series and 3.8% (3.9%) and 2.9% (3.0%) treated with EVT alone and with surgical reconstruction with/without EVT, respectively. The most common cause of death was cardiac disease, which accounted for 34% (34%) of all deaths. Postoperative complications were cardiac disease in 3.0% (3.0%), cerebrovascular disease in 1.8% (1.7%), pneumonia in 2.4% (2.4%), and wound complication in 4.5% (4.5%). Complications at the puncture site were noted in 1.0% (1.0%) of the limbs treated with EVT alone.

**Table:** Table 5 Outcomes early (one month) after treatment therapeutic measures: EVT (only EVT without surgical reconstruction), Surgical reconstruction (surgical reconstruction with or without EVT) Table 5-1 Life prognosis/causes of death

a. Total																
		Life prognosis			Causes of death											
		Alive	Dead	Intraoperative death	Cardiac disease	Cerebrovascular disease			Malignant neoplasm	Aortic aneurysm, dissection	Infection		Ischemic enteritis	Gastrointestinal bleeding	Others	Unknown
						Hemorrhage	Infarction	Unknown			Diseased limb	Others				
Local condition	Rutherford 4	137	3	0	2	(−)	0	0	0	0	0	1	0	0	0	0
	Rutherford 5	603	19	0	7	0	1	0	2	0	0	1	0	0	7	1
	Rutherford 6	114	10	0	2	0	0	0	0	0	0	3	1	0	4	0
Therapeutic measures	Non-reconstruction	48	4	0	0	0	0	0	0	0	0	2	0	0	2	0
	EVT	405	16	0	7	0	0	0	1	0	0	2	1	0	4	1
	Surgical reconstruction	401	12	0	4	0	1	0	1	0	0	1	0	0	5	0
	Total	854	32	0	11	0	1	0	2	0	0	5	1	0	11	1
b. ASO																
		Life prognosis			Causes of death											
		Alive	Dead	Intraoperative death	Cardiac disease	Cerebrovascular disease			Malignant neoplasm	Aortic aneurysm, dissection	Infection		Ischemic enteritis	Gastrointestinal bleeding	Others	Unknown
						Hemorrhage	Infarction	Unknown			Diseased limb	Others				
Local condition	Rutherford 4	136	3	0	2	0	0	0	0	0	0	1	0	0	0	0
	Rutherford 5	586	19	0	7	0	1	0	2	0	0	1	0	0	7	1
	Rutherford 6	112	10	0	2	0	0	0	0	0	0	3	1	0	4	0
Therapeutic measures	Non-reconstruction	46	4	0	0	0	0	0	0	0	0	2	0	0	2	0
	EVT	399	16	0	7	0	0	0	1	0	0	2	1	0	4	1
	Surgical reconstruction	389	12	0	4	0	1	0	1	0	0	1	0	0	5	0
	Total	834	32	0	11	0	1	0	2	0	0	5	1	0	11	1

EVT: endovascular treatment

**Table:** Table 5-2 Perioperative complications 1

a. Total																
		Cardiac disease				Cerebrovascular disease				Pneumonia		Wound complication		Peripheral embolism		
		(−)	Angina	Serious arrhythmia	Myocardial infarction	(−)	TIA	Cerebral infarction		(−)	(+)	(−)	(+)	(−)	(+)	
								Functional loss (−)	Functional loss (+)						Minor (including blue toe)	Major
Local condition	Rutherford 4	123	2	2	0	125	1	1	0	125	2	118	9	125	2	0
	Rutherford 5	578	10	3	2	584	0	4	5	579	14	571	22	586	6	1
	Rutherford 6	114	2	2	2	116	0	3	1	116	4	113	7	119	1	0
Therapeutic measures	Non-reconstruction	5	0	2	0	7	0	0	0	7	0	5	2	7	0	0
	EVT	412	6	0	3	416	0	2	3	406	15	413	8	412	8	1
	Surgical reconstruction	398	8	5	1	402	1	6	3	407	5	384	28	411	1	0
	Total	815	14	7	4	825	1	8	6	820	20	802	38	830	9	1
b. ASO																
		Cardiac disease				Cerebrovascular disease				Pneumonia		Wound complication		Peripheral embolism		
		(−)	Angina	Serious arrhythmia	Myocardial infarction	(−)	TIA	Cerebral infarction		(−)	(+)	(−)	(+)	(−)	(+)	
								Functional loss (−)	Functional loss (+)						Minor (including blue toe)	Major
Local condition	Rutherford 4	122	2	2	0	124	1	1	0	124	2	117	9	124	2	0
	Rutherford 5	563	10	3	2	569	0	4	5	564	14	557	21	571	6	1
	Rutherford 6	112	2	2	2	115	0	2	1	114	4	111	7	117	1	0
Therapeutic measures	Non-reconstruction	5	0	2	0	7	0	0	0	7	0	5	2	7	0	0
	EVT	406	6	0	3	410	0	2	3	400	15	407	8	406	8	1
	Surgical reconstruction	386	8	5	1	391	1	5	3	395	5	373	27	399	1	0
	Total	797	14	7	4	808	1	7	6	802	20	785	37	812	9	1

TIA: transient ischemic attack; EVT: endovascular treatment

**Table:** Table 5-3 Perioperative complications 2

a. Total															
		Hemorrhage			Site of bleeding			Outcome of bleeding				Complication due to contrast medium		Complication at puncture site	
		(−)	(+)	Unknown	Brain	GI tract	Others	Cured	Uncured	Dead	Others	(−)	(+)	(−)	(+)
Local condition	Rutherford 4	124	2	1	0	2	0	2	0	0	0	127	0	68	0
	Rutherford 5	581	11	1	0	4	7	8	1	2	0	590	3	352	3
	Rutherford 6	114	5	1	0	2	3	5	0	0	0	120	0	65	1
Therapeutic measures	Non-reconstruction	7	0	0	0	0	0	0	0	0	0	7	0	13	0
	EVT	415	5	1	0	2	3	5	0	0	0	419	2	417	4
	Surgical reconstruction	397	13	2	0	6	7	10	1	2	0	411	1	55	0
	Total	819	18	3	0	8	10	15	1	2	0	837	3	485	4
b. ASO															
		Hemorrhage			Site of bleeding			Outcome of bleeding				Complication due to contrast medium		Complication at puncture site	
		(−)	(+)	Unknown	Brain	GI tract	Others	Cured	Uncured	Dead	Others	(−)	(+)	(−)	(+)
Local condition	Rutherford 4	123	2	1	0	2	0	2	0	0	0	126	0	68	0
	Rutherford 5	567	10	1	0	4	6	7	1	2	0	575	3	345	3
	Rutherford 6	112	5	1	0	2	3	5	0	0	0	118	0	65	1
Therapeutic measures	Non-reconstruction	7	0	0	0	0	0	0	0	0	0	7	0	13	0
	EVT	409	5	1	0	2	3	5	0	0	0	413	2	411	4
	Surgical reconstruction	386	12	2	0	6	6	9	1	2	0	399	1	54	0
	Total	802	17	3	0	8	9	14	1	2	0	819	3	478	4

GI: gastrointestinal; EVT: endovascular treatment

**Table:** Table 5-4 Hemodynamics

a. Total													
		Immediate after the treatment						One month after the treatment					
		ABI		Ankle pressure		SPP		ABI		Ankle pressure		SPP	
		n	Median	n	Median	n	Median	n	Median	n	Median	n	Median
Local condition	Rutherford 4	75	0.83	64	107.0	35	38.0	68	0.83	59	110.0	27	40.0
	Rutherford 5	288	0.87	271	111.0	263	41.0	215	0.91	199	123.0	174	43.0
	Rutherford 6	36	0.85	36	114.5	25	42.0	31	0.89	32	116.5	27	43.0
Therapeutic measures	Non-reconstruction	21	0.84	17	110.0	16	35.0	11	0.86	9	110.0	8	33.5
	EVT	230	0.84	216	110.0	161	38.0	173	0.90	163	123.0	112	41.5
	Surgical reconstruction	148	0.86	138	113.0	146	44.5	130	0.88	118	113.0	108	42.5
	Total	399	0.85	371	111.0	323	41.0	314	0.89	290	119.5	228	41.5
b. ASO													
		Immediate after the treatment						One month after the treatment					
		ABI		Ankle pressure		SPP		ABI		Ankle pressure		SPP	
		n	Median	n	Median	n	Median	n	Median	n	Median	n	Median
Local condition	Rutherford 4	75	0.83	64	107.0	35	38.0	67	0.82	58	109.5	27	40.0
	Rutherford 5	281	0.86	266	112.0	257	41.0	212	0.91	196	123.5	171	43.0
	Rutherford 6	36	0.85	36	114.5	25	42.0	31	0.89	32	116.5	27	43.0
Therapeutic measures	Non-reconstruction	20	0.84	17	110.0	16	35.0	11	0.86	9	110.0	8	33.5
	EVT	225	0.84	212	110.5	158	38.0	172	0.91	162	123.0	112	41.5
	Surgical reconstruction	147	0.86	137	113.0	143	45.0	127	0.87	115	112.0	105	42.0
	Total	392	0.85	366	111.0	317	41.0	310	0.89	286	119.5	225	41.0

ABI: ankle brachial (pressure) index; SPP: skin perfusion pressure; EVT: endovascular treatment

**Table:** Table 5-5 Condition of the limbs

a. Total																		
		Bypass graft/EVT condition							Clinical symptom of the limb			Ischemic wound				Ambulatory function at discharge (Taylor’s classification)		
		Good	Stenosis	Occlusion	Deterioration	Anastomosis disruption (aneurysm)	Infection	Others	Improved	No change	Deteriorated	Cured	Uncured		Unknown	Ambulatory	Ambulatory/homebound	Nonambulatory
													Improved	Deteriorated				
Local condition	Rutherford 4	114	2	7	0	0	1	3	116	16	4	88	23	22	3	97	21	22
	Rutherford 5	535	16	31	0	1	0	9	494	101	17	143	349	117	3	330	140	152
	Rutherford 6	98	3	6	1	1	2	5	83	17	12	17	68	27	0	37	32	55
Therapeutic measures	Non-reconstruction	0	0	0	0	0	0	0	29	5	3	12	19	6	0	25	9	18
	EVT	372	17	15	1	0	1	15	315	77	22	102	209	100	3	207	75	139
	Surgical reconstruction	375	4	29	0	2	2	2	349	52	8	134	212	60	3	232	109	72
	Total	747	21	44	1	2	3	17	693	134	33	248	440	166	6	464	193	229
b. ASO																		
		Bypass graft/EVT condition							Clinical symptom of the limb			Ischemic wound				Ambulatory function at discharge (Taylor’s classification)		
		Good	Stenosis	Occlusion	Deterioration	Anastomosis disruption (aneurysm)	Infection	Others	Improved	No change	Deteriorated	Cured	Uncured		Unknown	Ambulatory	Ambulatory/homebound	Nonambulatory
													Improved	Deteriorated				
Local condition	Rutherford 4	113	2	7	0	0	1	3	115	16	4	88	22	22	3	96	21	22
	Rutherford 5	523	16	28	0	1	0	9	482	97	16	138	341	113	3	318	138	149
	Rutherford 6	96	3	6	1	1	2	5	81	17	12	17	66	27	0	37	31	54
Therapeutic measures	Non-reconstruction	0	0	0	0	0	0	0	28	4	3	11	18	6	0	25	9	16
	EVT	366	17	15	1	0	1	15	311	75	22	102	205	98	3	202	74	139
	Surgical reconstruction	366	4	26	0	2	2	2	339	51	7	130	206	58	3	224	107	70
	Total	732	21	41	1	2	3	17	678	130	32	243	429	162	6	451	190	225

EVT: endovascular treatment

**Table:** Table 5-6 Revision of treatment

a. Total																		
		Revision for those excluding good bypass graft/EVT condition		Minor reintervention (revision for stenosis)				Major reintervention (revision for occlusion)								Major amputation		
		(+)	(−)	(−)	Patch plasty	EVT	Others	(−)	Thrombectomy (±patch plasty)	Thrombolysis	EVT	Re-bypass	Jump bypass	Interposition	Others	(−)	(+)	
																	Due to preoperative sound	Due to new wound
Local condition	Rutherford 4	5	8	124	0	3	0	124	1	0	1	0	0	1	0	136	3	1
	Rutherford 5	31	26	568	2	14	3	559	4	0	5	11	5	1	2	599	14	3
	Rutherford 6	6	11	103	0	5	1	101	2	0	2	2	2	0	0	97	14	1
Therapeutic measures	Non-reconstruction	0	0	0	0	0	0	0	0	0	0	0	0	0	0	42	2	0
	EVT	23	26	395	1	15	3	398	1	0	4	7	3	1	0	393	18	3
	Surgical reconstruction	19	19	400	1	7	1	386	6	0	4	6	4	1	2	397	11	2
	Total	42	45	795	2	22	4	784	7	0	8	13	7	2	2	832	31	5
b. ASO																		
		Revision for those excluding good bypass graft/EVT condition		Minor reintervention (revision for stenosis)				Major reintervention (revision for occlusion)								Major amputation		
		(+)	(−)	(−)	Patch plasty	EVT	Others	(−)	Thrombectomy (±patch plasty)	Thrombolysis	EVT	Re-bypass	Jump bypass	Interposition	Others	(−)	(+)	
																	Due to preoperative sound	Due to new wound
Local condition	Rutherford 4	5	8	123	0	3	0	123	1	0	1	0	0	1	0	135	3	1
	Rutherford 5	29	25	554	2	14	2	547	3	0	5	11	4	1	1	584	13	2
	Rutherford 6	6	11	101	0	5	1	99	2	0	2	2	2	0	0	95	14	1
Therapeutic measures	Non-reconstruction	0	0	0	0	0	0	0	0	0	0	0	0	0	0	40	2	0
	EVT	23	26	390	1	15	2	392	1	0	4	7	3	1	0	388	17	3
	Surgical reconstruction	17	18	388	1	7	1	377	5	0	4	6	3	1	1	386	11	1
	Total	40	44	778	2	22	3	769	6	0	8	13	6	2	1	814	30	4

EVT: endovascular treatment

**Table:** Table 5-7 Condition of contralateral limbs

a. Total																		
		Contralateral limb occlusive lesions							Treatment for contralateral limb									
		(−)	(+)						Unnecessary	(+)								
			Asymptomatic	Intermittent claudication	CLI			Post treatment		Pharmacological therapy	Angiogenic therapy	EVT	Surgical bypass	Minor amputation	Major amputation	Lumbar sympathectomy	Necessary but no treatment	Others
					R4	R5	R6											
Local condition	Rutherford 4	47	52	6	2	2	1	25	8	63	0	15	9	1	4	0	0	0
	Rutherford 5	180	240	26	9	42	5	110	49	287	1	68	42	7	22	0	4	1
	Rutherford 6	30	41	3	2	5	6	34	10	54	1	12	14	1	10	0	4	1
Therapeutic measures	Non-reconstruction	16	14	0	0	0	1	18	2	13	0	13	4	2	6	0	0	0
	EVT	106	175	13	7	29	8	77	35	216	1	58	17	4	15	0	5	1
	Surgical reconstruction	135	144	22	6	20	3	74	30	175	1	24	44	3	15	0	3	1
	Total	257	333	35	13	49	12	169	67	404	2	95	65	9	36	0	8	2
b. ASO																		
		Contralateral limb occlusive lesions							Treatment for contralateral limb									
		(−)	(+)						Unnecessary	(+)								
			Asymptomatic	Intermittent claudication	CLI			Post treatment		Pharmacological therapy	Angiogenic therapy	EVT	Surgical bypass	Minor amputation	Major amputation	Lumbar sympathectomy	Necessary but no treatment	Others
					R4	R5	R6											
Local condition	Rutherford 4	47	52	6	2	2	1	24	8	63	0	15	8	1	4	0	0	0
	Rutherford 5	169	237	26	8	42	5	108	48	284	1	68	40	7	22	0	3	1
	Rutherford 6	28	41	3	2	5	6	34	10	54	1	12	14	1	10	0	4	1
Therapeutic measures	Non-reconstruction	14	14	0	0	0	1	18	2	13	0	13	4	2	6	0	0	0
	EVT	102	174	13	6	29	8	77	35	215	1	58	17	4	15	0	4	1
	Surgical reconstruction	128	142	22	6	20	3	71	29	173	1	24	41	3	15	0	3	1
	Total	244	330	35	12	49	12	166	66	401	2	95	62	9	36	0	7	2

CLI: critical limb ischemia; EVT: endovascular treatment

**Table:** Table 5-8 Malignant neoplasm

a. Total															
		Newly diagnosed malignant neoplasm			Sites of newly diagnosed malignant neoplasm										
		(−)	(+)	Unknown	Head and neck	Esophagus	Lung	Stomach	Hepatobiliary pancreas	Colon	Breast	Uterus	Ovarium	Prostate	Others
Local condition	Rutherford 4	138	1	1	0	1	0	0	0	0	0	0	0	0	0
	Rutherford 5	611	10	1	1	0	2	0	2	3	1	0	0	0	3
	Rutherford 6	123	0	1	0	0	0	0	0	0	0	0	0	0	0
Therapeutic measures	Non-reconstruction	50	0	2	0	0	0	0	0	0	0	0	0	0	0
	EVT	415	6	0	0	1	1	0	1	2	1	0	0	0	2
	Surgical reconstruction	407	5	1	1	0	1	0	1	1	0	0	0	0	1
	Total	872	11	3	1	1	2	0	2	3	1	0	0	0	3
b. ASO															
		Newly diagnosed malignant neoplasm			Sites of newly diagnosed malignant neoplasm										
		(−)	(+)	Unknown	Head and neck	Esophagus	Lung	Stomach	Hepatobiliary pancreas	Colon	Breast	Uterus	Ovarium	Prostate	Others
Local condition	Rutherford 4	137	1	1	0	1	0	0	0	0	0	0	0	0	0
	Rutherford 5	594	10	1	1	0	2	0	2	3	1	0	0	0	3
	Rutherford 6	121	0	1	0	0	0	0	0	0	0	0	0	0	0
Therapeutic measures	Non-reconstruction	48	0	2	0	0	0	0	0	0	0	0	0	0	0
	EVT	409	6	0	0	1	1	0	1	2	1	0	0	0	2
	Surgical reconstruction	395	5	1	1	0	1	0	1	1	0	0	0	0	1
	Total	852	11	3	1	1	2	0	2	3	1	0	0	0	3

EVT: endovascular treatment

The median ABI and SPP of the measured limbs, immediately after treatment and 1 month after treatment, were 0.85 (0.85) and 0.89 (0.89) and 41 (41) mmHg and 41.5 (41) mmHg, respectively. Stenosis, occlusion, infection, or other trouble occurred after revascularization by EVT alone in 11.6% (11.8%) and by surgical reconstruction with/without EVT in 9.4% (9.0%). The rate of secondary major amputation was 5.1% (4.9%) in EVT alone and 3.2% (3.0%) in surgical reconstruction with/without EVT. When ambulatory function at discharge was compared with that before surgery, the rate of ambulatory patients changed from 60% (59%) to 52% (52%), ambulatory/homebound patients from 20% (20%) to 22% (22%), and non-ambulatory patients from 21% (21%) to 26% (26%).

The problems, comments, and considerations on these spreadsheets are described below. The number of “bypass graft/EVT condition,” “clinical limb symptoms,” “ischemic wound,” and “ambulatory function at discharge” did not match (**Table 5-5**). The total number of “ambulatory function at discharge” was 886 (866), which was equal to the number of life prognoses (**Table 5-1**), indicating no “unused.” The number of “bypass graft/EVT condition” was not equal to the number of “ambulatory function at discharge” because the objective of “bypass graft/EVT condition” was to achieve survival with arterial reconstruction of the limbs and because more than one condition could be selected. The numbers of “clinical symptoms of limb” and “ischemic wound” were not identical. They must be identical because their objective was to achieve survival without major amputations. This is speculated to be due to the presence of “unused” in dead cases before registration. The discrepancy in the total number of “life prognosis,” “clinical limb symptom,” and “amputation” is due to the difference of condition for data aggregation. In **Table 5-3**, the presence of the puncture site complication in non-reconstruction group seems to be odd. The registration of complication at the puncture site was required in limbs where PTA/STENT was selected in the revascularization method. However, in JCLIMB, multiple treatment methods other than revascularization were selected, which caused the odd results. It is presumed to be due to input error or EVT failure.

The number of limbs of survivors with EVT was 405 (399 limbs) (**Table 5-1**), which was 9 (9) limbs less than the sum of the number in the column of minor reintervention or major reintervention in the row of limbs with EVT, 414 limbs (408 limbs) (**Table 5-6**). The number of limbs of survivors with surgical reconstruction was 401 (389 limbs) (**Table 5-1**), which was 8 (8) limbs less than the sum of the number in the column of minor reintervention or major reintervention in the row of limbs with surgical reconstruction, 409 limbs (397 limbs) (**Table 5-6**). This is speculated to be due to death after reintervention. In **Table 5-6**, the objective for input of “revision for those excluding good bypass graft/EVT condition” is limb registered in stenosis, occlusion, deterioration, anastomosis disruption (aneurysm), infection, and others of “bypass graft/EVT condition.” The number of “bypass graft/EVT condition” of surgical reconstruction and total in **Table 5-5** does not match the number of “revision for those excluding good bypass graft/EVT condition” in **Table 5-6**. This is because multiple items can be selected in “bypass graft/EVT condition,” and both “infection” and “anastomosis disruption (aneurysm)” were selected in a case. The total number of “the contralateral limb occlusive lesions” in **Table 5-7** is 18 limbs (18 limbs) less than “life prognosis” in **Table 5-1** due to missing values. The sum of the number of “treatment for contralateral limb” is less than that of “the contralateral limb occlusive lesions” as the objectives of “treatment for contralateral limb” excluded the limbs with no occlusive lesions in the contralateral limb. Because multiple registrations were possible, the sum of the number of “treatment for contralateral limb” was more than that of the limbs with occlusive lesions in the contralateral limb. When a patient died within 1 month, the information of “newly diagnosed malignant neoplasm” at death was registered in **Table 5-8**.

In addition to the above, there were some parts where the total number does not match in **Tables 5-1** to **5-8**. It might be because several items had multiple choices or missing values.

## 4. Conclusions

Vascular surgeons’ contribution to the participating facilities is the sufficient amount of detailed data during busy clinical practice, which has gradually elucidated the current status of CLI treatment in Japan. Data on CLI in 2018 were elucidated, after the annual data in 2013–2017. The JCLIMB Committee is planning to continue publishing an annual report in the future. In 2017, the new concept, “chronic limb threatening ischemia,” was proposed instead of CLI,^14)^ and a new clinical guideline, the Global Vascular Guideline, was published instead of TASC in 2019.^15)^ The full name of JCLIMB has been changed to “Japan Chronic Limb-Threatening Ischemia Database,” and the data format has been revised to register the survey items according to the Global Vascular Guideline, which can be used in 2021.

The JCLIMB Committee expects that these study results will be fed back to clinical situations to help develop medical care for CLI. The paper regarding 30 days’ prediction model using the data of JCLIMB has been published,^16)^ and the paper regarding 2 years’ prediction model was submitted. Facilities can participate in JCLIMB at any time by contacting the JSVS secretariat for details.

● “CLI” was used in this paper because the objectives registered in 2019 were based on CLI defined by TASC II.

## 5. Participant Facilities (83 Facilities in the Order of the Japanese Syllabary by Prefecture, Corporate Names are Omitted as a Rule)

Department of Vascular Surgery, Asahikawa Medical University Hospital

Department of Cardiovascular Surgery, National Hospital Organization Obihiro Hospital

Department of Cardiovascular Surgery, Nayoro City General Hospital

Department of Cardiovascular Surgery, Hirosaki University Hospital

Department of Surgery, Iwate Prefectural Isawa Hospital

Department of Surgery, Iwate Prefectural Chubu Hospital

Department of Vascular Surgery, Morioka Yuai Hospital

Department of Surgery, JR Sendai Hospital

Department of Cardiovascular Surgery, Sendai City Hospital

Department of Transplantation, Reconstruction and Endoscopic Surgery, Tohoku University Hospital

Department of Cardiovascular Surgery, Saiseikai Yamagata Saisei Hospital

Department of Vascular and Endovascular Surgery, Ibaraki Prefectural Central Hospital

Department of Cardiac and Vascular Surgery, Dokkyo Medical University Hospital

Department of Vascular and Endovascular Surgery, International University of Health and Welfare

Department of Vascular Surgery, Saiseikai Kawaguchi General Hospital

Department of Vascular Surgery, Saitama Medical Center, Saitama Medical University

Department of Cardiovascular Surgery, Saitama Medical Center, Jichi Medical University

Department of Cardiovascular Surgery, Jichi Medical University

Department of Surgery, Saitama City Hospital

Department of Cardiovascular Surgery, Shimada General Hospital

Department of Cardiovascular Surgery, Chiba Cerebral and Cardiovascular Center

Department of Cardiovascular Surgery, Itabashi Chuo Medical Center

Department of Cardiovascular Surgery, IMS Tokyo Katsushika General Hospital

Department of Surgery, Edogawa Hospital

Department of Surgery, Tokyo Metropolitan Health and Medical Treatment Corporation, Okubo Hospital

Department of Cardiovascular Surgery, Kyorin University

Department of Surgery, Keio University School of Medicine

Department of Vascular Surgery, International University of Health and Welfare, Mita Hospital

Department of Vascular Surgery, Tokyo Medical and Dental University

Department of Cardiovascular Surgery, Tokyo Medical University Hachioji Medical Center

Department of Cardiovascular Surgery, Tokyo Medical University Hospital

Department of Vascular Surgery, The Jikei University Kashiwa Hospital

Department of Vascular Surgery, The Jikei University Hospital

Department of Vascular Surgery, The University of Tokyo Hospital

Department of Cardiovascular Surgery, Tokyo Rinkai Hospital

Department of Vascular Surgery, Nihon University Itabashi Hospital

Department of Surgery, Shonankamakura General Hospital

Department of Cardiovascular Surgery, St. Marianna University School of Medicine

Department of Surgery, Tomei Atsugi Hospital

Department of Cardiovascular Surgery, Yokosuka General Hospital Uwamachi

Department of Cardiovascular Surgery, National Hospital Organization, Kanazawa Medical Center

Department of Cardiovascular Surgery, Shizuoka Red Cross Hospital

Department of Surgery II, Yamanashi University Hospital

Department of Vascular Surgery, Aichi Medical University Hospital

Department of Vascular Surgery, Ichinomiya Municipal Hospital

Department of Vascular Surgery, Japanese Red Cross Nagoya Daiichi Hospital

Department of Vascular Surgery, Nagoya University Hospital

Department of Vascular Surgery, Osaka Rosai Hospital

Department of Vascular Surgery, Aijinkai Inoue Hospital

Department of Vascular Surgery, Nippon Life Hospital

Department of Vascular Surgery, Kansai Medical University Medical Center

Department of Cardiovascular Surgery, Toyonaka Municipal Hospital

Department of Cardiovascular Surgery, Suita Tokushukai Hospital

Department of Cardiovascular Surgery, Tsukazaki Hospital

Department of Cardiovascular Surgery, Kobe University Hospital

Department of Thoracic and Cardiovascular Surgery, Wakayama Medical University Hospital

Department of Cardiovascular Surgery, Tottori Prefectural Kousei Hospital

Department of Cardiovascular Surgery, Tottori Prefectural Central Hospital

Department of Cardiovascular Surgery, Okayama University Hospital

Department of Cardiovascular Surgery, Kawasaki Medical School General Medical Center

Department of Cardiovascular Surgery, Kawasaki Medical School Hospital

Department of Cardiovascular and Respiratory Surgery, Hiroshima Prefectural Hospital

Department of Cardiovascular Surgery, National Hospital Organization, Higashihiroshima Medical Center

Department of Cardiovascular Surgery, Hiroshima University Hospital

Department of Surgery, Saiseikai Yamaguchi General Hospital

Department of Surgery 1, Yamaguchi University Hospital

Department of Cardiovascular Surgery, Ehime Prefectural Central Hospital

Department of Cardiovascular Surgery, Matsuyama Shimin Hospital

Department of Vascular Surgery, Matsuyama Red Cross Hospital

Department of Cardiovascular Surgery, Kochi Health Sciences Center

Department of Cardiovascular Surgery, Kochi University Hospital

Department of Vascular Surgery, National Hospital Organization, Kyushu Medical Center

Department of Surgery and Science, Kyushu University Hospital

Department of Cardiovascular Surgery, Kurume University Hospital

Department of Vascular Surgery, Kokura Memorial Hospital

Department of Surgery, Saiseikai Fukuoka General Hospital

Department of Surgery, Saiseikai Yahata General Hospital

Department of Vascular Surgery, Fukuoka City Hospital

Department of Vascular Surgery, National Hospital Organization, Fukuokahigashi Medical Center

Department of Surgery, Saiseikai Karatsu Hospital

Department of Cardiovascular Surgery, Sasebo Chuo Hospital

Department of Vascular Surgery, Kumamoto Rehabilitation Hospital

Department of Cardiovascular Surgery, Oita Oka Hospital

## 6. JCLIMB Committee, NCD JCLIMB Analytical Team

### (1) JCLIMB Committee

Shinsuke Mii (Chairman), Kunihiro Shigematsu (Vice Chairman), Nobuyoshi Azuma, Atsuhisa Ishida, Yoshinori Inoue, Hisashi Uchida, Takao Ohki, Sosei Kuma, Koji Kurosawa, Michinari Kono, Akio Kodama, Hiroyoshi Komai, Kimihiro Komori, Takashi Shibuya, Shunya Shindo, Ikuo Sugimoto, Juno Deguchi, Katsuyuki Hoshina, Hirofumi Midorikawa, Terutoshi Yamaoka, Hiroya Yamashita, and Yasuhiro Yunoki, and Tetsuro Miyata (Observer)

### (2) NCD JCLIMB Analytical Team

Arata Takahashi and Hiroaki Miyata

## Additional Remarks

This report was authorized by the institutional review board of Saiseikai Yahata General Hospital. (Authorization No.185)

## Additional Note

The original Annual Report was published in Japanese Journal of Vascular Surgery Vol. 31 (2022) No. 3; however, errors in numerical data were detected after the publication. The errata were published in Vol. 31 (2022) No. 5 of the same journal. This translation reflects that correction.

## Notes

This is a translation of Jpn J Vasc Surg 2022; 31: 157–185.

## References

[1] Japanese Society for Vascular Surgery JCLIMB Committee, NCD JCLIMB Analytical Team. 2013 JAPAN Critical Limb Ischemia Database (JCLIMB) Annual Report. Ann Vasc Dis 2016; 9: 356-73.28018515

[2] Japanese Society for Vascular Surgery JCLIMB Committee, NCD JCLIMB Analytical Team. 2014 JAPAN Critical Limb Ischemia Database (JCLIMB) Annual Report. Ann Vasc Dis 2016; 9: 374-91.28018516

[3] Japanese Society for Vascular Surgery JCLIMB Committee, NCD JCLIMB Analytical Team. 2015 JAPAN Critical Limb Ischemia Database (JCLIMB) Annual Report. Ann Vasc Dis 2018; 11: 398-426. 30402196 10.3400/avd.ar.18-00048 PMC6200626

[4] Japanese Society for Vascular Surgery JCLIMB Committee, NCD JCLIMB Analytical Team. 2016 JAPAN Critical Limb Ischemia Database (JCLIMB) Annual Report. Ann Vasc Dis 2019; 12: 109-35. 30931073 10.3400/avd.ar.19-00012 PMC6434344

[5] Japanese Society for Vascular Surgery JCLIMB Committee, NCD JCLIMB Analytical Team. 2017 JAPAN Critical Limb Ischemia Database (JCLIMB) Annual Report. Ann Vasc Dis 2020; 13: 205-33. 32595803 10.3400/avd.ar.20-00018 PMC7315241

[6] Japanese Society for Vascular Surgery JCLIMB Committee, NCD JCLIMB Analytical Team. 2018 JAPAN Critical Limb Ischemia Database (JCLIMB) Annual Report. Ann Vasc Dis 2021; 14: 202-30. 34239652 10.3400/avd.ar.21-00047 PMC8241553

[7] Norgren L, Hiatt WR, Dormandy JA, et al. Inter-society consensus for the management of peripheral arterial disease (TASC II). J Vasc Surg 2007; 45 **Suppl S**: S5-67. 17223489 10.1016/j.jvs.2006.12.037

[8] Mills JL Sr, Conte MS, Armstrong DG, et al. The Society for Vascular Surgery Lower Extremity Threatened Limb Classification System: risk stratification based on wound, ischemia, and foot infection (WIfI). J Vasc Surg 2014; 59: 220-34.e2. 24126108 10.1016/j.jvs.2013.08.003

[9] Japanese Society of Nephrology. Clinical Practice Guideline for the Evaluation and Management of Chronic Kidney Disease 2012. Tokyo: Tokyo Igakusya; 2012. (in Japanese)

[10] Taylor SM, Kalbaugh CA, Gray BH, et al. The LEGS score: a proposed grading system to direct treatment of chronic lower extremity ischemia. Ann Surg 2003; 237: 812-9; discussion, 818-9. 12796577 10.1097/01.SLA.0000071563.19208.50 PMC1514690

[11] Armstrong DG, Lavery LA, Harkless LB. Validation of a diabetic wound classification system. The contribution of depth, infection, and ischemia to risk of amputation. Diabetes Care 1998; 21: 855-9. 9589255 10.2337/diacare.21.5.855

[12] Yamada T, Ohta T, Ishibashi H, et al. Clinical reliability and utility of skin perfusion pressure measurement in ischemic limbs—comparison with other noninvasive diagnostic methods. J Vasc Surg 2008; 47: 318-23. 18241755 10.1016/j.jvs.2007.10.045

[13] Rutherford RB, Baker JD, Ernst C, et al. Recommended standards for reports dealing with lower extremity ischemia: revised version. J Vasc Surg 1997; 26: 517-38. 9308598 10.1016/s0741-5214(97)70045-4

[14] Aboyans V, Ricco JB, Bartelink MLEL, et al. 2017 ESC guideline on the diagnosis and treatment of peripheral arterial diseases, in collaboration with the European Society of Vascular Surgery (ESVS): document covering atherosclerotic disease of extracranial carotid and vertebral, mesenteric, renal, upper and lower extremity arteries. Eur Heart J 2018; 39: 763-816. 28886620 10.1093/eurheartj/ehx095

[15] Conte MS, Bradbury AW, Kolh P, et al. Global vascular guidelines on the management chronic limb-threatening ischemia. J Vasc Surg 2019; 69 **6S**: 3S-125S, e40. 31159978 10.1016/j.jvs.2019.02.016 PMC8365864

[16] Miyata T, Mii S, Kumamaru H, et al. Risk prediction model for early outcomes of revascularization for chronic limb-threatening ischaemia. Br J Surg 2021; 108: 941-50. 33693591 10.1093/bjs/znab036

